# Improvements in the Appearance and Nutritional Quality of Tomato Fruits Resulting from Foliar Spraying with Silicon

**DOI:** 10.3390/foods13020223

**Published:** 2024-01-10

**Authors:** Li Wang, Ning Jin, Yandong Xie, Wen Zhu, Ye Yang, Jiaying Wang, Yongzhong Lei, Wenkai Liu, Shuya Wang, Li Jin, Jihua Yu, Jian Lyu

**Affiliations:** 1 College of Horticulture, Gansu Agriculture University, Lanzhou 730070, China; 17752210335@163.com (L.W.); jinn0513@163.com (N.J.); xieyandongsx@163.com (Y.X.); 18394155589@163.com (W.Z.); yangye991022@163.com (Y.Y.); 17899315930@163.com (J.W.); leiyongzhong1023@163.com (Y.L.); 18093668927@163.com (W.L.); yujihuagg@163.com (J.Y.); 2 State Key Laboratory of Aridland Crop Science, Gansu Agricultural University, Lanzhou 730070, China; wsyhn95@163.com (S.W.); jinli0124@163.com (L.J.)

**Keywords:** tomato, silicon, quality, firmness, carotenoids, amino acids

## Abstract

Research on silicon (Si), an element considered beneficial for plant growth, has focused on abiotic and biotic stress mitigation. However, the effect of Si on tomato fruit quality under normal growth conditions remains unclear. This study investigated the effects of applying different levels of Si (0 mmol·L^−1^ [CK], 0.6 mmol·L^−1^ [T1], 1.2 mmol·L^−1^ [T2], and 1.8 mmol·L^−1^ [T3]) in foliar sprays on tomato fruit quality cultivated in substrates, and the most beneficial Si level was found. Compared to CK, exogenous Si treatments had a positive influence on the appearance and nutritional quality of tomato fruits at the mature green, breaker, and red ripening stages. Of these, T2 treatment significantly increased peel firmness and single-fruit weight in tomato fruits. The contents of soluble sugars, soluble solids, soluble proteins, and vitamin C were significantly higher, and the nitrate content was significantly lower in the T2 treatment than in the CK treatment. Cluster analysis showed that T2 produced results that were significantly different from those of the CK, T1, and T3 treatments. During the red ripening stage, the a* values of fruits in the T2 treatment tomato were significantly higher than those in the other three treatments. Moreover, the lycopene and lutein contents of the T2 treatment increased by 12.90% and 17.14%, respectively, compared to CK. T2 treatment significantly upregulated the relative gene expression levels of the phytoene desaturase gene (*PDS*), the lycopene *ε*-cyclase gene (*LCY-E*), and the zeaxanthin cyclooxygenase gene (*ZEP*) in the carotenoid key genes. The total amino acid content in tomato fruits in the T2 treatment was also significantly higher than that of CK. In summary, foliar spraying of 1.2 mmol·L^−1^ exogenous Si was effective in improving the appearance and nutritional quality of tomato fruits under normal growth conditions. This study provides new approaches to further elucidate the application of exogenous silicon to improve tomato fruit quality under normal conditions.

## 1. Introduction

The tomato (*Solanum lycopersicum* L.), belonging to the genus Solanaceae, is an annual or perennial herb consumed by many people. It contains beneficial vitamin C, lycopene, amino acids, carbohydrates, organic acids, and other nutrients [1]. Global tomato production reached 186,821 million tons in 2020 from a planted area spanning 5,051,983 ha [2]. As consumer demand for tomatoes increases, the tomato cultivation area expands annually [3]. Tomato quality is determined by both intrinsic inner qualities (such as flavor and nutritional value) and external qualities (such as size and color), which are key factors in determining consumer acceptance and recognition [4]. Improving the quality of tomatoes can not only increase consumers’ preference for the fruit but also improve the economic benefits of tomatoes as well [5]. The growth, yield, and fruit quality of tomatoes are influenced by genetic factors, environmental factors, and cultivation measures [6,7]. Unfortunately, when it comes to production, growers pursue higher yields blindly, including excessive use of chemical fertilizers, pesticides, and other unfavorable inputs, resulting in a decline in the quality of tomatoes. Improving the quality of tomatoes is particularly important for production [8,9]. Presently, tomatoes are generally believed to have lost their “childhood taste” [10]. Therefore, many researchers are focusing on improving the quality of tomatoes.

Silicon (Si) is the second most abundant element in the soil after oxygen and is considered a quasi-essential element in higher plants [11]. Si can be used as a biostimulant in horticultural production, applied to plants through foliar sprays, and incorporated in soil or fertigation applications [12]. Previous studies have shown that cereals, such as grasses and sedges are rich in Si, which is stored in vacuoles and cell walls. Si can enhance the strength of plant cell walls and promote the development of mechanical tissues in plants [13]. Numerous studies have reported the positive effects of exogenous Si on plant growth and development, particularly under stressful conditions. For instance, when facing high boron levels, adding 2 mmol·L^−1^ sodium silicate to the nutrient solution can mitigate the adverse effects on tomato growth and yield [14]. Similarly, spraying cucumber foliage with 2.25 mmol·L^−1^ potassium silicate during drought and salinity stress can enhance cucumber fruit firmness and delay fruit senescence [15]. Furthermore, under water deficit conditions, including 2.5 mmol·L^−1^ potassium silicate in the nutrient solution can boost the chlorophyll and carotenoid contents in tomato leaves effectively [16]. In drought stress, the foliar application of 20% silicic acid at 200 and 400 kg·ha^−1^ can have a positive impact on the flesh thickness and total soluble solid content in cantaloupe fruits [17]. Under water deficit conditions, foliar application of 2.8 g·L^−1^ Si to tomato plants increased the net photosynthesis assimilation rate of leaves, stomatal conductance, and the transpiration rate; improved gas exchange; and promoted photosynthesis [18]. Under salt stress, adding 2.0 mmol·L^−1^ potassium silicate to the nutrient solution effectively increased the concentration of soluble sugars and starch in Cabernet Sauvignon grapevines, thereby enhancing their salt tolerance [19]. When subjected to water stress, using potassium silicate significantly reduced the transpiration rate of strawberries, improved plant water-use efficiency, and mitigated the adverse effects of water stress on strawberry plants [20]. When rice blast occurs in early spring, applying 300 kg· ha^−1^ silicon dioxide (SiO_2_) reduces the damage caused by rice pests [21].

Si plays an essential role in plant growth, yield, and quality [22]. Jarosz [23] showed that applying 100.0 mg·dm^−3^ of SiO_2_ in a tomato nutrient solution significantly increased tomato fruit yield. Analogically, root application of 500 and 750 mg·dm^−3^ of Si to cucumbers effectively enhances the total soluble solids content and yield of cucumber fruits [24]. Additionally, foliar spraying of apples with 20 kg·hm^2^ SiO_2_ has been found to promote the accumulation of total phenolic and anthocyanin compounds in the various apple tissues [25]. Pati et al. [26] found that the application of 600 kg·ha^−1^ of Si can increase rice yield. Currently, studies on Si have mainly focused on abiotic stressors [27], including drought [28], autotoxicity [29], and salt [30] stress, and on biotic stressors, including Phytophthora in soybeans [31], melons and wheat powdery mildew [32,33], rice borers and brown planthoppers [34,35], and wheat with alate aphids [36], among others. However, the effects of Si on the appearance and nutritional quality of tomatoes under normal conditions have rarely been reported. Hence, the objective of this study is to examine the effects of foliar spraying of different levels of Si on primary and secondary metabolism using high-performance liquid chromatography (HPLC) and liquid chromatograph mass spectrometry (LC-MS) of tomato fruits. Our study was designed to screen for suitable Si concentrations and to provide new insights into the use of Si to improve tomato fruit appearance and nutritional quality by foliar spraying suitable concentrations of silicon under normal growth conditions.

## 2. Materials and Methods

### 2.1. Plant Materials

The experiment was conducted in a modern greenhouse (36.10° N, 103.72° E) at the experimental base of the College of Horticulture, Gansu Agricultural University, Anning District, Lanzhou City, Gansu Province, between March 2021 and October 2021. The tomato seeds (cv. ‘Jingfan 502’) used in this experiment were purchased from the Gansu Academy of Agricultural Sciences, China. The Si used was provided by the chemical reagent sodium silicate (Na_2_SiO_3_·9H_2_O, AR, Yuan ye Biotechnology Co. Ltd., Shanghai, China).

### 2.2. Experimental Design

**Figure 1:**
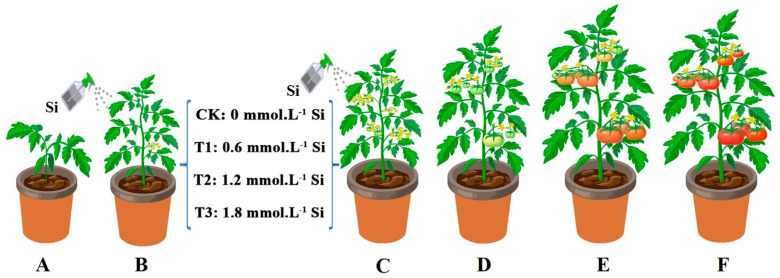
The experimental treatment setup and sampling schematic illustration. (**A**) Seedlings; (**B**) foliar spraying starts; (**C**) end of foliar spraying; (**D**) mature green stage sampling; (**E**) breaker stage sampling; (**F**) red ripening stage sampling.

The experiment was conducted with seedlings in an artificial climate box (RDN-400E-4; Ningbo Dongnan Instrument Co. Ltd., Zhejiang, China) at the Gansu Agricultural University. When the tomato seedlings grew to the three-leaf, one-heart stage, they were colonized in the modern greenhouse of Gansu Agricultural University, where the average annual temperature was 13 °C. The experiment utilized substrate cultivation with grass charcoal (vermiculite: pearl substrate ratio of 2:1:1). The substrate exhibited a maximum field water holding capacity of 61.03%. The basic physicochemical properties of the substrates are presented in Supplementary Table S1. The colonization pots had a height of 20 cm and a diameter of 26 cm. From the blooming of the first tomato fruit spike, foliar spraying of exogenous Si treatment was performed with a foliar sprayer at 9:00 a.m. every 7 days until the blooming of the fifth fruit spike, such that the foliage was uniformly wet to the same degree. On the same day, the blossoming fruits were tagged and labeled, and the other management practices were consistent. The experiment was conducted with four treatments, and each treatment was repeated three times, with 25 pots per replication. The experimental treatment setup and sampling are shown in Figure 1. The pH (6.8 ± 0.1) of each treatment solution was adjusted with 1 mol·L^−1^ H_2_SO_4_.

### 2.3. Experimental Methods

#### 2.3.1. Indicators of Appearance Quality

The longitudinal and transverse diameters of the tomato fruits were measured using Vernier calipers and a fruit shape index (longitudinal diameter/transverse diameter) [37]. A single fruit was weighed on an electronic balance. The fruit color parameters, including lightness (L*), redness (a*), and yellowness (b*), were measured using a colorimeter (CR-10 Plus; Konica Minolta, Inc., Tokyo, Japan). The firmness of the fruit peel was determined using a GY-4-J digital fruit firmness tester (Top Cloud-Agri Technology, Hangzhou, China) [38].

#### 2.3.2. Indicators of Nutritional Quality

The soluble sugar content of the tomato fruits was measured using the anthrone-sulfuric acid colorimetric method [39]. The soluble solid content was measured using a portable handheld refractometer PAL-1 (Atago, PR-1, Atago Co. Ltd., Tokyo, Japan) [40]. The soluble protein content was assessed using the Coomassie Brilliant Blue G-250 staining method [41]. The vitamin C content was determined using the 2,6-dichlorophenol indophenol staining method [42]. The nitrate content was determined using the salicylic acid–sulfuric acid colorimetric method [43].

#### 2.3.3. Determination of Carotenoid Components

Extraction and detection of carotenoid components were performed according to the method described by Wang et al. [38], with some modifications. Frozen tomato fruit powder (0.5 g) was added to 30 mL of a mixture of petroleum ether and acetone. The mixture was then subjected to ultrasonic extraction for 40 min. The solution was then transferred to a separatory funnel, filtered, rinsed thrice with ultrapure water to drain the aqueous phase, and poured into a triangular flask. Anhydrous sodium sulfate (1 g) was added, and the liquid was transferred from the triangular flask to a round-bottomed flask, and evaporated with a rotary evaporator for 2 min. The resulting solution was dissolved in a mixture of acetonitrile, dichloromethane, and methanol (25 mL). Subsequently, the extracts from the wall of the bottle were dissolved and filtered through a double-layer organic filtration membrane of 0.22 µm into the brown sample bottles. The filtrate was analyzed using high-performance liquid chromatography (HPLC Waters, Milford, MA, USA). External standard method was used for quantitative analysis in quality control (QC). Five tomato fruit pigments were detected: phytoene, lycopene, β-carotene, lutein, and violaxanthin. The chromatographic conditions were as follows: the chromatography column was C18 (250 mm × 4.6 mm, 5 µm, Waters Symmetry, USA), and the detection wavelength was 472 nm. The flow rate was 1.2 mL·min^−1^. The mobile phase was conducted with methanol, acetonitrile, and dichloromethane (25:55:20, *v*/*v*/*v*), and the column temperature was 30 °C. Based on the response time of each compound wavelength and the peak area of the standard sample (Shanghai Yuan Ye Biotechnology Co., Ltd. Shanghai, China), a standard curve function was created, and quantitative calculations were carried out. Each treatment was set up in three replicates, and the data were analyzed using Empower software (version 3.7.0; Waters, MA, USA).

#### 2.3.4. Expression of Key Genes in Relation to Carotenoid Metabolic Pathways

The expression levels of key enzyme genes in relation to the carotenoid metabolic pathway were determined, including the phytoene synthase*1* gene (*PSY1*), phytoene desaturase gene (*PDS*), lycopene*β*-cyclase gene (*LCY-B*), lycopene *ε*-cyclase gene (*LCY-E*), and zeaxanthin cyclooxygenase gene (*ZEP*). Total RNA was extracted from tomato fruits using a Tiangen RNAprep Pure Plant Kit, and cDNA synthesis was performed using a Tiangen Fastking RT Kit (Tiangen Biotech, Co., Ltd., Beijing, China). The experimental procedures were performed strictly according to the manufacturer’s instructions. The design of the quantitative fluorescence primers (Sangon Biotech Co., Ltd., Shanghai, China) is presented in Table 1. The tomato *Actin* gene was used as an internal reference gene, and a quantitative reverse transcription polymerase chain reaction was performed using a real-time fluorescence quantitative polymerase chain reaction instrument (Roche, Rotkreuz, Switzerland). Each sample was repeated thrice. The relative expression levels of each sample and control were calculated using the 2^−ΔΔCT^ method.

#### 2.3.5. Determination of Amino Acid Components

Extraction and detection of amino acid components were performed as described by Nimbalkar et al. [44], with some modifications. Accurately weighed 0.1 g frozen tomato fruit powder was combined with 1 mL of 0.5 M hydrochloric acid aqueous solution for extraction. The samples were mixed at 6600× *g* for 20 min using a vortex mixer (MX-S, Scilogex, San Diego, CA, USA) and then extracted by ultrasound at 25 °C for 20 min. After ultrasonication, the samples were centrifuged (318KS, Sigma, Osterode am Harz, Germany) at 20,000× *g* for 20 min. The supernatant was filtered through a double-layer 0.22 µm aqueous filter into a brown sample vial for quantitative analysis by HPLC-mass spectrometry (LC-MS, Agilent 1290-6460, Santa Clara, CA, USA). External standard method was used for quantitative analysis in quality control (QC). The HPLC parameters were set as follows: The chromatographic column used, Agilent Infinity Lab Poroshell 120 HILIC-Z (2.1 × 100 mm, 2.7 μm), was prepared with 200 mmol·L^−1^ ammonium formate stock solution with water, and its pH was adjusted to 3 with formic acid. Mobile phase A was a water/ammonium formate stock solution (9:1), and mobile phase B (acetonitrile) was an ammonium formate stock solution (9:1). The flow rate was 0.5 mL/min. The column temperature was 25 °C. Based on the response time of each compound wavelength, the peak area of a standard sample purchased from Merck and Sigma (Sigma-Aldrich GmbH, Sternheim, Germany) was used as the standard curve function, and quantitative calculations were performed.

### 2.4. Statistical Analysis

Data were organized using Microsoft Excel 2010 software (Microsoft Inc., Redmond, WA, USA). SPSS software (version 22.0; SPSS Institute Inc., Chicago, IL, USA) was used for one-way analysis of variance, and Duncan’s multiple range test (*p* < 0.05) was used to compare significant differences between treatments. The results are expressed as mean ± standard error (SE). In all analyses, a probability value below 0.05 was considered statistically significant (*p* < 0.05). Principal component analysis (PCA) and cluster analysis via a heat map with a dendrogram were performed using Origin 2021 (Origin, Inc., San Francisco, CA, USA).

## 3. Results

### 3.1. Effects of Different Levels of Si on the Appearance Quality of Tomato Fruits

**Figure 2:**
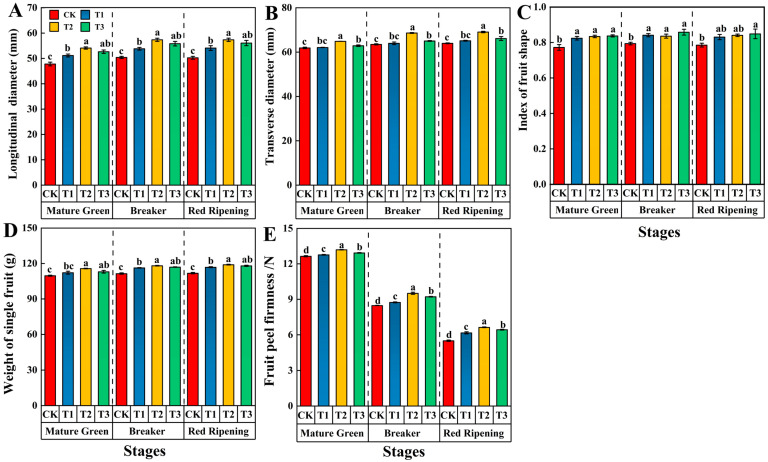
Effects of different levels of Si on the longitudinal diameter (**A**), transverse diameter (**B**), fruit shape index (**C**), single fruit weight (**D**), and fruit peel firmness (**E**) in tomato fruit during the mature green, breaker and red ripening stages. Data are presented as the mean ± SE of three replicates of each sample. Different lowercase letters indicate significant differences according to Duncan’s multiple range tests (*p* < 0.05). Abbreviations: CK: 0 mmol·L^−1^ Si; T1: 0.6 mmol·L^−1^ Si; T2: 1.2 mmol·L^−1^ Si; T3: 1.8 mmol·L^−1^ Si.

The appearance quality of tomato fruits was affected by different levels of silicon. The longitudinal diameter of tomato fruits first increased and then decreased with increasing Si levels. The longitudinal diameter of tomato fruits was significantly increased by 13.22%, 13.83%, and 14.15% in the T2 treatment compared to CK at the mature green, breaker, and red ripening stages, respectively (Figure 2A). The transverse diameter of tomato fruits in the three stages increased and then decreased with increasing exogenous Si concentrations. In the mature green, breaker, and red ripening stages, the transverse diameter of tomato fruits in the T2 treatment significantly improved by 4.83%, 8.08%, and 7.96%, respectively, compared to those of CK (Figure 2B). Regarding the fruit shape index, the T3 treatment produced results significantly higher than CK by 8.42%, 8.06%, and 8.15% at the mature green, breaker, and red ripening stages, respectively (Figure 2C). In the T2 treatment, the single fruit weight increased significantly by 5.55%, 5.98%, and 6.45% over CK at the three stages, respectively (Figure 2D). As tomato fruits ripen, the rind gradually softens. The peel firmness of tomato fruits increased and then decreased with increasing Si concentration at the mature green, breaker, and red ripening stages. Peel firmness of the tomato fruits treated with T2 was the largest, showing significant increases of 4.35%, 12.28%, and 20.55% compared to CK (Figure 2E).

### 3.2. Effects of Different Levels of Si on the Nutritional Quality and Safety Quality of Tomato Fruits

**Figure 3:**
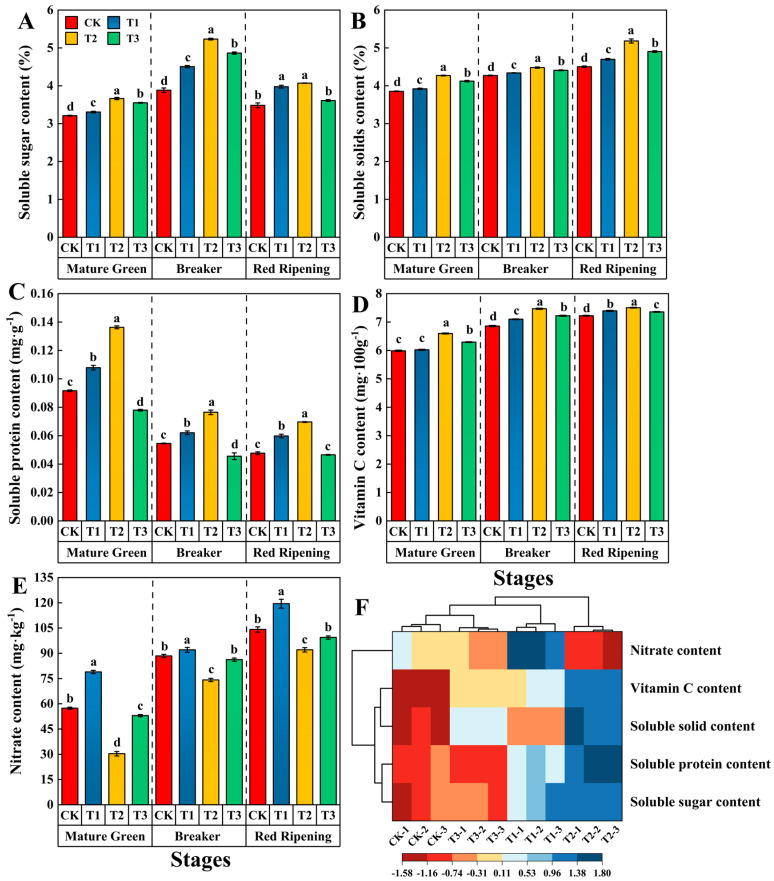
Effects of different levels of Si on the nutritional quality and safety quality in tomato fruits during the mature green, breaker and red ripening stages. (**A**) Soluble sugars; (**B**) soluble solids; (**C**) soluble protein; (**D**) vitamin C; (**E**) nitrate content; (**F**) cluster analysis. Data are presented as the mean ± SE of three replicates of each sample. Different lowercase letters indicate significant differences according to Duncan’s multiple range tests (*p* < 0.05). Abbreviations: CK: 0 mmol·L^−1^ Si; T1: 0.6 mmol·L^−1^ Si; T2: 1.2 mmol·L^−1^ Si; T3: 1.8 mmol·L^−1^ Si.

Si treatment enhanced the soluble sugar content of tomato fruits at the mature green, breaker, and red-ripening stages. Specifically, the mature green and breaker stages of the T2 treatment were significantly higher than those of the CK, T1, and T3 treatments. Compared to CK, the soluble sugar content of fruits undergoing the T2 treatment was significantly increased by 14.02%, 34.79%, and 16.95% at the mature green, breaker, and red ripening stages, respectively (Figure 3A). Applying Si treatments resulted in an increase in the soluble solids content of tomato fruits at the mature green, breaker, and red ripening stages, with the T2 treatment producing significant improvements of 10.91%, 4.92%, and 15.11% compared to CK. The differences between the four treatments were significant (Figure 3B). After Si treatment, the soluble protein content increased and then decreased with tomato fruit ripening. Compared to CK, the soluble protein content in fruits treated with T2 increased by 47.83%, 40.74%, and 45.83% at the mature green, breaker, and red ripening stages, respectively (Figure 3C). The vitamin C content of tomato fruits increases during ripening, and the vitamin C content of tomato fruits treated with Si was higher than that of CK. At the mature green and breaker stages, tomato fruits treated with T2 had vitamin C content that was 10.18% and 8.89% higher than those treated with CK, respectively. In the red ripening stage, the vitamin C content of tomato fruits treated with T2 reached the maximum value of 7.51 mg·100 g^−1^, which was a significant increase of 4.01% compared to CK (Figure 3D). The nitrate content of tomato fruits tended to increase and then decrease with increasing Si levels. Notably, T2 treatment significantly reduced the nitrate content in tomato fruits. At the mature green, breaker, and red ripening stages, the T2 treatment resulted in a decrease of 47.17%, 16.12%, and 11.58%, respectively, compared to CK (Figure 3E). The classification model, based on cluster analysis, classified the four treatments into three large categories: one for the CK and T3 treatments, one for the T1 treatment, and another for the T2 treatment (Figure 3F).

### 3.3. Effects of Different Levels of Si on the Color Parameters and Carotenoid Metabolism of Tomato Fruits

#### 3.3.1. Effect of Different Levels of Si on the Color Parameters of Tomato Fruits

**Figure 4:**
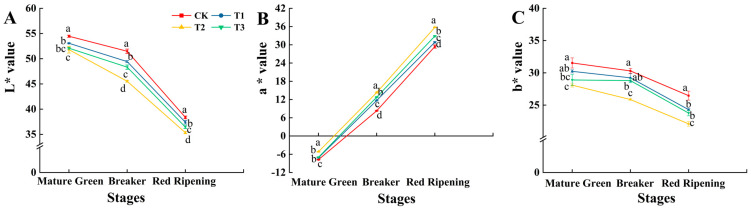
Effects of different levels of Si on the L* (**A**), a* (**B**), and b* (**C**) in tomato fruits during the mature green, breaker, and red ripening stages. Data are presented as the mean ± SE of three replicates of each sample. Different lowercase letters indicate significant differences according to Duncan’s multiple range tests (*p* < 0.05). Abbreviations: CK: 0 mmol·L^−1^ Si; T1: 0.6 mmol·L^−1^ Si; T2: 1.2 mmol·L^−1^ Si; T3: 1.8 mmol·L^−1^ Si.

Different levels of exogenous Si treatment had different degrees of promoting effects on the ripening coloration of tomato fruits. With the ripening of tomato fruits, the color parameters L* and b* of tomato fruits treated with the T1, T2, and T3 treatments showed a gradual downward trend, whereas a* showed a gradual upward trend. The color parameters L* and b* of tomato fruits treated with T1, T2, and T3 were lower than those treated with CK, while a* was higher than that of CK (Figure 4A–C). Particularly, the T2 treatment produced levels of L* that were significantly lower than those produced by CK at the mature green, breaker, and red ripening stages, with decreases of 4.90%, 11.58%, and 7.92%, respectively (Figure 4A). The T2-treated fruits’ a* was significantly increased by 33.46%, 72.29%, and 21.54% compared to CK at the mature green, breaker, and red ripening stages (Figure 4B). The T2-treated fruits’ b* was significantly lower than that of fruits treated with CK at the mature green, breaker, and red ripening stages, with decreases of 10.88%, 14.62%, and 16.51%, respectively (Figure 4C). Throughout the growth stage, the fruit color parameters L* and b* of fruit treated with T2 were lower than those of the other three treatments, whereas a* was higher (Figure 4).

#### 3.3.2. Effects of Different Levels of Si on the Carotenoid Components of Tomato Fruits

Changes were found in the carotenoid components in red-ripening-stage tomato fruits with different levels of Si (Table 2). Specifically, the phytoene content in the T1 treatment showed an increase of 0.66% compared to CK. Lycopene content showed an increasing and then decreasing trend with increasing Si concentration and was significantly increased by 12.90% as a result of the T2 treatment compared to CK. Compared to CK, the levels of β-carotene in fruits treated with T1 and of lutein in fruits treated with T2 were significant increased by 7.18% and 17.14%, respectively. Compared to CK, the content of violaxanthin in fruits treated with T2 showed a reduction of 4.18%, whereas no significant difference was observed between the T2 and T3 treatments. The classification model based on cluster analysis can be classified into two large categories: one for the CK and T1 treatments and the other for the T2 and T3 treatments.

#### 3.3.3. Effects of Different Levels of Si on the Key Gene Expression of Carotenoid Metabolism Pathway in Tomato Fruits

**Figure 5:**
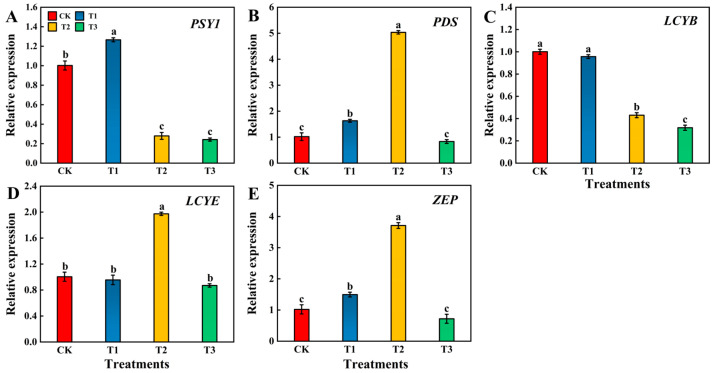
Effects of different levels of Si on the key enzyme gene relative expression of carotenoid metabolism pathway of tomato fruits at the red ripening stage. (**A**) *PSY1*; (**B**) *PDS*; (**C**) *LCYB*; (**D**) *LCYE*; (**E**) *ZEP*. Data are presented as the mean ± SE of three replicates of each sample. Different lowercase letters indicate significant differences according to Duncan’s multiple range tests (*p* < 0.05). Abbreviations: CK: 0 mmol·L^−1^ Si; T1: 0.6 mmol·L^−1^ Si; T2: 1.2 mmol·L^−1^ Si; T3: 1.8 mmol·L^−1^ Si.

At the red ripening stage of tomato fruits, the T2 treatment significantly increased the gene expression of *PDS*, *LCYE*, and *ZEP*. Compared to CK, the *PSY1* expression produced by the T1 treatment was significantly upregulated by 26.35%, and that produced by the T2 and T3 treatments was significantly downregulated by 72.16% and 75.85%, respectively (Figure 5A). *PDS* gene expression was significantly upregulated by 393.63% in fruits treated with T2 compared to CK, whereas no significant difference was observed between the CK and T3 treatments (Figure 5B). *LCYB* gene expression produced by the T2 and T3 treatments was significantly downregulated by 569% and 682% compared to CK, respectively, and no significant difference was observed between the CK and T1 treatments (Figure 5C). *LCYE* gene expression was significantly upregulated by 96.42% in fruits treated with T2 compared to CK. Additionally, the gene expression level was significantly upregulated by 106.49% and 126.63% in fruits treated with T2 compared to those treated with the T1 and T3 treatments (Figure 5D). Compared to CK, the *ZEP* gene expression produced by the T2 treatment was significantly upregulated by 263.37%, and no significant difference was observed between the CK and T3 treatments (Figure 5E).

### 3.4. Effects of Different Levels of Si on the Amino Acid of Tomato Fruits

**Figure 6:**
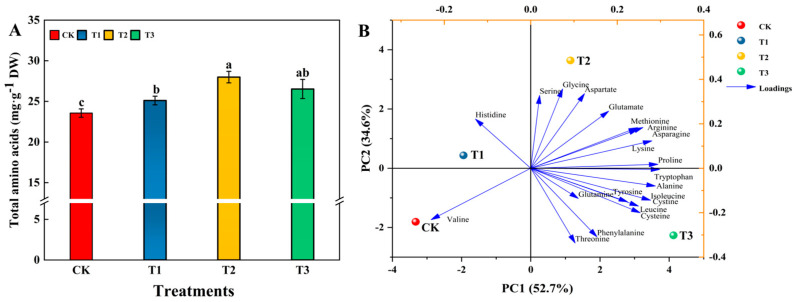
Effects of different levels of Si on the amino acid of tomato fruits at the red ripening stage. (**A**) Total amino acid; (**B**) PCA. Data are presented as the mean ± SE of three replicates of each sample. Different lowercase letters indicate significant differences according to Duncan’s multiple range tests (*p* < 0.05). Abbreviations: CK: 0 mmol·L^−1^ Si; T1: 0.6 mmol·L^−1^ Si; T2: 1.2 mmol·L^−1^ Si; T3: 1.8 mmol·L^−1^ Si.

A total of 21 free amino acids from the red ripening stage of tomato fruits were measured using LC-MS (Figure 6). Among them, compared to CK, methionine and aspartic acid were significantly increased by 20.33% and 68.18%, respectively, in fruits treated with T2, and proline was significantly increased by 71.15% in fruits treated with T3 (Supplementary Materials and Table S2). Different levels of Si treatment significantly increased the total amino acid content of tomato fruits compared to that of CK. Of these, the total amino acids in fruits treated with T2 were the most significant, increasing by 18.79% compared to CK and by 11.46% and 5.51% compared to the T1 and T3 treatments, respectively (Figure 6A). PCA was performed on the amino acid components at different concentrations of Si. PCA of the tomato fruit amino acid components showed that the first two principal components explained 87.3% of the total variance, whereas PC1 and PC2 contributed 52.7% and 34.6% of the variance, respectively. From the loading diagram, it was observed that proline and tryptophan have strong first principal component loadings, and serine, histidine, and glycine have strong second principal component loadings; hence, they were used as representative factors for reacting to different concentrations of exogenous silica on tomato amino acids (Figure 6B).

## 4. Discussion

In tomato production, fruit shape plays an essential role in economic outcomes, determining the main use of a particular variety, and is a key element in consumer choice and purchase [45]. A tomato’s economic value is determined by both yield and quality. Fresh fruit weight is an important external quality indicator for tomatoes. The tomato fruit yield can be expressed in terms of the number and weight of fruits [5]. Compared to cucumber plants only treated with water, applying Si fertilizer to cucumber through foliar spraying and soil increased the weight and number of cucumber fruits by 2.1 kg·m^−2^ and 23 fruits·m^−2^, respectively [46]. Abidi et al. [47] also observed that the composite spraying of 3% (300 cc·100 L^−1^) and 4.5% (450 cc·100 L^−1^) potassium Si fertilization on the foliage of peaches increased the single fruit weight and soluble solid content, improved flesh firmness, and reduced the malic acid content of the fruits. Foliar spraying of potassium silicate at 3 cm^3^·L^−1^ on potatoes significantly increased the weight of tubers per plant and the average weight of tubers [48]. Foliar spray of silicic acid at 2 and 4 mL·L^−1^ increased both the grain and straw yield of rice production [49]. These studies suggest that leaves can absorb silicic acid directly and/or stimulate a plant to absorb more nutrients [50]. Our experimental results indicate that applying exogenous Si can effectively improve the fruit shape index and single fruit weight of tomato fruits, with the fruit shape index of the T3 treatment and the single fruit weight of the T2 treatment showing the most significant effects.

After the tomato fruit ripens, the peel and fruit gradually soften, resulting in cracking, metamorphosis, rot, and other phenomena during the transportation process, which seriously affects the commercial value of the fruit and results in a substantial loss of commercial and economic value [51]. Firmness plays a major role in determining post-harvest quality. It prolongs shelf life by changing the tightness or thickness of the fruit cell wall. Consequently, enhancing fruit firmness and facilitating fruit harvesting are essential for modern agricultural development. Si is a significant component of cell walls and increases their mechanical strength (firmness) and stability. Si can also improve the thickness and firmness of the fruit peel and increase fruit toughness, thus improving the cell’s resistance to pressure and its tensile ability [13]. Both root and foliar application of 75 mg·L^−1^ potassium silicate to capsicum plants increased the cuticle thickness and the firmness of the peppers, probably because of the increase in the mechanical strength of the fruits caused by the Si treatment [52]. Foliar spraying with 2 mmol·L^−1^ SiO_2_ also significantly increased fruit firmness at the end of cucumber peduncles under saline soil conditions [53]. In our study, we also found that spraying different concentrations of exogenous Si on tomato leaf surfaces increased the firmness of the tomato fruit rind. This could be because Si is a component of the cell wall, and applying exogenous Si can increase the Si content in the cell wall of the fruit peel. Accordingly, applying exogenous Si to increase the firmness of tomato fruit rind provides great convenience for long-distance transportation and extends the shelf life [54].

The quality of tomatoes is the most important factor in the production process, and improving the quality of tomatoes can actively promote the production of tomato fruits. Ascorbic acid has a strong antioxidant effect and is an important nutrient for determining tomato quality [55]. The flavor, taste, and water content of fruits are influenced by soluble solids. Hence, increasing the content of soluble solids in fruits can enhance the quality and value of fresh tomatoes [56]. Some studies have found that the foliar application of 0.6 g·L^−1^ potassium silicate to strawberry plants can increase the vitamin C and total soluble solids content of strawberries [57]. Field experiments in Poland have shown that applying exogenous Si can increase the vitamin C content in potato tubers and significantly reduce the nitrate content [58]. Foliar spraying of Si on tomatoes can effectively increase the content of fruit-soluble solids and ascorbic acid and enhance the fruit’s firmness [59]. Stamatakis et al. [60] also observed that potassium silicate could increase the firmness and vitamin C content of tomato fruits. Foliar sprays of 5 mmol·L^−1^ potassium silicate increased leaf chlorophyll and soluble sugar content in potatoes [61]. Consistent with our results, we found that soluble solids, vitamin C, soluble sugars, and soluble proteins were increased, and nitrate content was reduced in exogenous Si-treated tomato fruits, all of which were best treated with 1.2 mmol·L^−1^ of Si.

Tomato fruits are rich in vitamin A, ascorbic acid, other vitamins, proteins, potassium, phytosterols (β-sitosterol, campesterol and stigmasterol), and folic acid and contain a large number of phytochemicals, such as phenolic compounds and carotenoids [62,63]. Carotenoids (lycopene, β-carotene, lutein, zeaxanthin, and β-cryptoxanthin) have attracted great interest in the field of human nutrition, where they act as biological antioxidants that contribute to the organism’s defense against reactive oxygen species. Additionally, carotenoids play a crucial protective role in major diseases, such as diabetes and cardiovascular diseases [64]. Fruit color is an important and complex attribute of fruit quality and another important factor influencing consumer preference for tomatoes [65]. The color of tomato fruits continues to deepen during growth and development, mainly because of chlorophyll degradation and gradual carotenoid accumulation [66]. Carotenoid biosynthesis involves a series of reactions, including condensation, dehydrogenation, cyclization, hydroxylation, and epoxidation [67]. Carotenoid biosynthesis begins with condensing two geranylgeranyl diphosphate molecules by *PSY* to form the 15-*cis*-isomer phytoene. Phytoene is converted to lycopene through a series of desaturated isomerization reactions. Lycopene is cyclized by β-LCY and ε-LCY or β-LCY to form α-carotene or β-carotene. These carotenes are further hydroxylated to produce lutein (for example, lutein and zeaxanthin) [68]. The carotenoid synthesis pathway has become a target of transgenic studies to modify the expression of key genes, such as *PSY*, *PDS,* and *LCY-β* [69]. Our results suggested that the color parameters L* and b* of tomato fruits treated with Si were lower than those of fruits treated with CK, whereas a* was higher than that of fruits treated with CK, indicating that foliar spraying of exogenous Si accelerated the ripening of tomato fruits and promoted color change. Stamatakis et al. [60] found that under salt stress, adding Si to a nutrient solution of tomatoes could significantly increase the content of lycopene and β-carotene in tomato fruits. In this study, we also found that the T2 treatment increased lycopene and lutein content in tomato fruit. Additionally, the T2 treatment significantly upregulated the relative expression levels of *PDS*, *LCYE,* and *ZEP* compared to CK in the carotenoid key genes.

**Figure 7:**
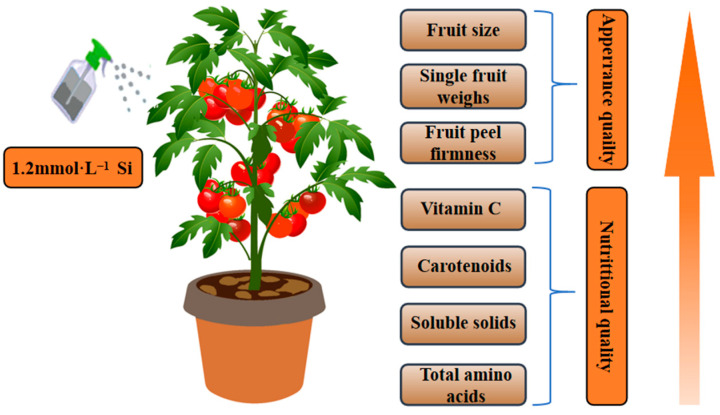
Potential mechanism enacted by foliar spraying of 1.2 mmol·L^−1^ Si to improve fruit appearance and nutritional quality by altering the primary and secondary metabolism of tomatoes.

Amino acids are important nitrogen-containing substances in fruits and vegetables and are the basic components of proteins. The flavor amino acids in fruits and vegetables are sweet (alanine, glycine, and serine), sour (aspartate and glutamate), and bitter (leucine, phenylalanine, tryptophan, and tyrosine). Amino acids are closely related to human taste and play an important role in the flavor and nutritional value of fruits and vegetables [70,71]. Under alkaline stress, the total free amino acid content of corn seedlings can be significantly increased by injecting 1.5 mmol·L^−1^ sodium metasilicate into sterilized corn seeds [72]. The application of Si fertilizer (80% orthosilicic acid) in soil could increase the content of essential amino acids (threonine, isoleucine, and leucine) and non-essential amino acids (aspartate, glutamate, serine, alanine, tyrosine, arginine, and proline) in Shengdao 14 and Huaidao 11 rice cultivars [73]. In this study, foliar spraying of Si effectively increased the content of aspartate and total amino acids in tomato fruits, which may be similar to the above study and help improve the flavor of tomato fruits, with the effects of the T2 treatment being most significant. Foliar spraying of 1.2 mmol·L^−1^ Si may improve fruit appearance and nutritional quality by altering the primary and secondary metabolism of tomatoes (Figure 7).

## 5. Conclusions

In this study, foliar spraying of exogenous Si had a positive effect on tomato fruit quality under normal growth conditions. Among them, foliar spraying of 1.2 mmol·L^−1^ (T2) Si treatment was the most effective. This was attributed to the fact that the addition of Si improved the nutritional quality of tomato by significantly increasing the soluble sugars, soluble solids, soluble proteins, and vitamin C contents, promoting the accumulation of carotenoids (lycopene and lutein), and increasing the total amino acid content of tomato fruits. Therefore, this study provides theoretical support for future research on high-quality cultivation of tomatoes.

## Figures and Tables

**Table 1:** Primer sequences for qRT-PCR (quantitative reverse transcription polymerase chain reaction) analysis of genes related to carotenoid metabolism pathway.

Gene Name	Accession Number	Forward Primer (5′–3′)	Reverse Primer (5′–3′)
*Actin*	Solyc11g005330	TGTCCCTATTTACGAGGGTTATGC	CAGTTAAATCACGACCAGCAAGAT
*PSY1*	NM_001247883	CTGGTACGGTTGGGTTGATGAGTG	CGGATAGACTGCCTGTGCTAATTC
*PDS*	NM_001247166	CCCATGCCACGACCAGAAGATTG	TGCTGTAGACAAACCACCCAAACC
*LCYB*	NM_001247297	GAGTCGTTGGAATCGGTGGTACAG	AGAAGCCATGCCAATAACGAGGTC
*LCYE*	NM_001247408	TCCTGCTGGTCTTGCTCTTGC	TCTTTGAACTCGTCCTCCCATACAC
*ZEP*	NM_001309304	AAGAGGTCGTGTTACATTGCTTGG	ATGATTGCAGCCATTCTAGCCAGC

Abbreviations: *PSY:* phytoene synthase*1* gene; *PDS*: phytoene desaturase gene, *LCY-B*: lycopene *β*-cyclase gene; *LCY-E:* lycopene *ε*-cyclase gene; *ZEP:* zeaxanthin cyclooxygenase gene.

**Table 2:** Effect of different levels of Si on the carotenoid components of tomato fruits at the red ripening stage.

Treatments	CK	T1	T2	T3
Phytoene content (ug·g^−1^)	10.508 ± 0.077 a	10.584 ± 0.126 a	9.980 ± 0.146 b	8.956 ± 0.223 c
Lycopene content (mg·g^−1^)	6.822 ± 0.129 b	6.835 ± 0.179 b	7.703 ± 0.126 a	6.895 ± 0.111 b
β-carotene content (mg·g^−1^)	6.410 ± 0.012 b	6.872 ± 0.089 a	6.033 ± 0.031 c	6.064 ± 0.079 c
Lutein content (mg·g^−1^)	0.347 ± 0.003 c	0.359 ± 0.003 c	0.407 ± 0.004 a	0.393 ± 0.005 b
Violanthin content (ug·g^−1^)	28.451 ± 0.069 ab	29.096 ± 0.692 a	27.262 ± 0.085 bc	26.147 ± 0.465 c

Note: The data are expressed as average values ± SE. Different lowercase letters indicate significant differences according to Duncan’s multiple range tests (*p* < 0.05). Abbreviations: CK: 0 mmol·L^−1^ Si; T1: 0.6 mmol·L^−1^ Si; T2: 1.2 mmol·L^−1^ Si; T3: 1.8 mmol·L^−1^ Si.

## Data Availability

Data is contained within the article or Supplementary Material.

## Supplementary Materials

The following supporting information can be downloaded at: https://www.mdpi.com/article/10.3390/foods13020223/s1, Table S1: Basic physical and chemical properties of substrates; Table S2: Effect of different levels of silicon on the content of amino acid components (mg·g^−1^ DW) in tomato fruits; Table S3: RSD (Relative standard deviation), Recovery rate, LOD (Limit of detection), and LOQ (Limit of quantitation) of carotenoid by HPLC; Table S4: RSD (Relative standard deviation), Recovery rate, LOD (Limit of detection), and LOQ (Limit of quantitation) of amino acids determined by LC-MS.
