# Supplementary material for: Improvements in the Appearance and Nutritional Quality of Tomato Fruits Resulting from Foliar Spraying with Silicon
Source: Foods. 2024 Jan 10;13(2):223. doi: 10.3390/foods13020223 (PMC10814949; doi:10.3390/foods13020223)
Supplement: Supplementary file 1 [file foods-13-00223-s001.zip › foods-2804666-supplementary.pdf]

Supplementary materials

Table S1 Basic physical and chemical properties of substrates

substrates bulk density (kg·m^{-3})	pH	EC (mS·cm^{-1})	total nitrogen (g·kg^{-1})	alkaline hydrolyzed nitrogen (mg·kg^{-1})	available phosphorus (mg·kg^{-1})	available potassium (mg·kg^{-1})
521.860	7.800	2.100	1.612	498.600	136.700	346.500

Table S2 Effect of different levels of silicon on the content of amino acid components (mg·g^{-1} DW) in tomato fruits

Amino acid type	Treatments			
	CK	T1	T2	T3
Phenylalanine	0.223±0.014ab	0.213±0.007b	0.212±0.007b	0.234±0.013a
Threonine	0.215±0.010ab	0.203±0.009ab	0.200±0.006b	0.222±0.015a
Tryptophan	0.736±0.008c	0.767±0.021c	0.798±0.040ab	0.844±0.020a
Leucine	0.482±0.017b	0.489±0.004ab	0.485±0.008ab	0.505±0.010a
Isoleucine	0.486±0.010b	0.487±0.010b	0.492±0.005b	0.514±0.010a
Methionine	0.305±0.007c	0.333±0.008b	0.367±0.016a	0.360±0.022ab
Tyrosine	0.030±0.001b	0.028±0.001c	0.030±0.001b	0.032±0.001a
Valine	0.071±0.001a	0.069±0.003a	0.067±0.001a	0.068±0.001a
Proline	0.052±0.001c	0.066±0.010b	0.075±0.007b	0.089±0.002a
Cysteine	0.040±0.001a	0.033±0.002a	0.042±0.001a	0.148±0.186a
Alanine	0.302±0.020c	0.332±0.011bc	0.345±0.004b	0.413±0.029a
Glutamate	8.285±0.271c	8.207±0.234c	10.455±0.247a	9.162±0.674b
Glycine	0.131±0.018a	0.138±0.005a	0.145±0.027a	0.134±0.023a
Glutamine	7.640±0.228b	8.712±0.720a	7.663±0.318b	8.670±0.402a
Lysine	0.750±0.022b	0.752±0.031b	0.885±0.071a	0.851±0.040a
Serine	0.199±0.027a	0.223±0.015a	0.226±0.031a	0.205±0.033a
Asparagine	0.081±0.005a	0.086±0.003a	0.091±0.523a	0.092±0.016a
Aspartate	2.222±0.129c	2.695±0.096b	3.737±0.110a	2.667±0.182b
Histidine	0.870±0.076a	0.831±0.010a	0.883±0.042a	0.815±0.028a
Arginine	0.352±0.030b	0.356±0.016b	0.428±0.028a	0.408±0.029a
Cystine	0.090±0.006a	0.089±0.002a	0.090±0.006a	0.091±0.003a
Total amino acids	23.561±0.515c	25.109±0.533b	27.987±0.711[a]	26.525±1.177[ab]

Note: The data are expressed as average values ± SE. Different lowercase letters indicate significant differences according to Duncan's multiple range tests ($p < 0.05$). Abbreviations: CK: 0 mmol·L^{-1} Si; T1: 0.6 mmol·L^{-1} Si; T2: 1.2 mmol·L^{-1} Si; T3: 1.8 mmol·L^{-1} Si.

Table S3 RSD (Relative standard deviation), Recovery rate, LOD (Limit of detection), and LOQ (Limit of quantitation) of carotenoid by HPLC.

Carotenoid	RSD (%)	Recovery rate (%)	LOD /(ug·g)	LOQ /(ug·g)
Phytoene	4.52	95.53	0.10	0.33
Lycopene	3.94	90.28	0.58	1.20
β- carotene	4.40	92.91	0.15	0.43
Lutein	5.51	93.64	0.15	0.45
violaxanthin	3.15	95.86	0.20	0.62

Abbreviations: RSD: Relative Standard Deviation; LOD: Limit of detection; LOQ: Limit of quantitation.

Table S4 RSD (Relative standard deviation), Recovery rate, LOD (Limit of detection), and LOQ (Limit of quantitation) of amino acids determined by LC-MS.

Amino acids	RSD (%)	Recovery rate (%)	LOD ($ng·mL^{-1}$)	LOQ ($ng·mL^{-1}$)
Phenylalanine	2.93	103.23	0.53	1.71
Threonine	4.14	95.90	0.86	2.91
Tryptophan	3.96	96.25	1.09	3.60
Leucine	2.86	97.42	0.70	2.28
Isoleucine	2.58	94.86	0.65	2.24
Methionine	3.32	98.11	1.01	3.41
Tyrosine	2.78	96.17	3.32	11.03
Valine	2.33	96.18	1.39	4.57
Proline	1.99	97.40	0.59	1.93
Cysteine	2.89	96.31	1.07	3.96
Alanine	3.62	98.48	1.15	3.83
Glutamate	3.75	95.65	0.81	2.76
Glycine	4.51	103.40	0.78	2.62
Glutamine	3.16	95.72	0.88	2.82
Lysine	5.66	101.01	0.26	0.80
Serine	4.65	96.84	0.63	2.15
Asparagine	2.95	94.23	3.01	10.03
Aspartate	3.85	96.21	0.83	2.78
Histidine	3.74	97.69	1.86	6.23
Arginine	3.66	95.37	0.70	2.37
Cystine	3.64	91.5	1.14	3.74

Abbreviations: RSD: Relative Standard Deviation; LOD: Limit of detection; LOQ: Limit of quantitation.
